# Infant-derived *Bifidobacterium* strains screened *in vitro* for alleviating intestinal disorder caused by *Escherichia coli*

**DOI:** 10.3389/fnut.2026.1788810

**Published:** 2026-05-15

**Authors:** Wenqing Lv, Chonghua Fan, Zhenye Shi, Shuang Li, Mengna Zhao, Haixin Jiang, Guanyu Tao, Bailiang Li

**Affiliations:** 1 State Key Laboratory of Food Science and Resources, Jiangnan University, Wuxi, China; 2 Key Laboratory of Dairy Science, Ministry of Education, Northeast Agricultural University, Harbin, China

**Keywords:** antibacterial mechanism, bifidobacterium, gut microbiota, intestinal disorder, probiotic properties

## Abstract

**Introduction:**

*Escherichia coli* (*E. coli*) is a common foodborne pathogen that can cause various infections such as diarrhea and meningitis.

**Methods:**

In this study, 38 *Bifidobacterium* strains isolated from healthy breastfed infants were evaluated *in vitro*. D2, which demonstrated the strongest antagonism against *E. coli* and the most favorable probiotic properties, was selected for further investigation *in vivo*. The acid removal experiment of the cell-free supernatant (CFS) of D2 was conducted. Animal experiments were also performed, comparing D2 with A14 (a strain showing no antibacterial effect *in vitro*).

**Results:**

Acetic acid was identified as the main antibacterial substance in the CFS of D2 (*P* < 0.05). The CFS of D2 inhibited *E. coli* through multiple mechanisms, including inhibiting biofilm formation, damaging the integrity of the *E. coli* cell wall/membrane, and causing content leakage, ultimately leading to bacterial death. Furthermore, D2 significantly reduced the adhesion and invasion of E. coli on HT29 cells (*P* < 0.05). Compared with A14, D2 significantly reversed colonic damage caused by *E. coli*, restored cytokine expression, and promoted the expression of tight junction proteins. Additionally, D2 effectively ameliorated *E. coli*-induced gut microbiota dysbiosis and restored microbial diversity. It significantly reduced the abundance of Escherichia and promoted the abundance of beneficial bacterial genera such as Lachnospiraceae_NK4A136_group in the intestines.

**Discussion:**

These results indicate that D2 inhibits *E. coli* through multiple target points and represents a potential candidate strain for alleviating intestinal disorders caused by *E. coli*, or an alternative to antibiotics.

## 1 Introduction

Common foodborne pathogens include *Escherichia coli* (*E. coli*), *Staphylococcus aureus* (*S. aureus*), and *Salmonella*. The foodborne diseases caused by these pathogens can pose serious threats to human health (1). Acute infectious diarrhea is regarded as the second most common cause of death among children in developing countries, surpassed only by acute respiratory infections, accounting for approximately 20% of all child deaths (2). Enterotoxigenic *E. coli* is estimated to cause approximately 1.5 million deaths annually, ranking it among the most lethal foodborne pathogens worldwide (3). *E. coli* mainly produces either one or both of two types of enterotoxins, namely heat-labile enterotoxin

(LT) and heat-stable enterotoxin (ST) (4). The two enterotoxins have similar structures and both have a molecular weight of 84 kDa. The active subunit is surrounded by five subunits (5). The formation of biofilms is regarded as one of the characteristics of *E. coli*. Their biofilms are considered to be structured aggregates formed by bacterial cells embedded in extracellular polymeric substances (EPS), and their main components include DNA, proteins, polysaccharides and water (6). It can protect bacteria and facilitate the spread of foodborne diseases (7). Due to the formation of biofilms and the increasing antibiotic resistance of bacteria, the treatment of bacterial infections has become more challenging. Therefore, there is an urgent need to find a safe and healthy treatment method.

The sterile gastrointestinal tract of the infant is quickly occupied by microorganisms from the mother's feces, vagina, skin and the surrounding environment after birth (8). During the first few weeks of life, newborns rely on their innate immune system, antibodies transferred from the mother during pregnancy, as well as molecules and cells in breast milk, to defend against microbial invasion and establish effective interaction with the constantly developing microbial community of the infant (9). The initial intestinal microbiota was mainly composed of the Actinobacteria phylum and the Proteobacteria phylum. Later, with the introduction of the Firmicutes phylum and the Bacteroidetes phylum, it became more diverse (10). After the initial colonization is completed, the microbial community of healthy full-term breastfed infants will rapidly transform into a composition dominated by the Actinobacteria phylum (especially the *Bifidobacterium* genus) (11). The colonization of *bifidobacteria* is partly attributed to their strong preference for human milk oligosaccharides (12). They can even reach over 70% of the intestinal microbial community during breastfeeding (13).

Currently, the research on the screening of superior *bifidobacteria* and their inhibitory effect on *E. coli* has received extensive attention. The intestinal microbiota of infants is a rich source of *bifidobacteria*, and its abundance gradually decreases with the increase of age. *Bifidobacteria* are regarded as a common type of intestinal probiotics, possessing multiple benefits, which can improve the balance of intestinal microbiota and be beneficial to the host's health; they can inhibit the growth of pathogenic bacteria, directly suppressing intestinal pathogens by lowering the intraluminal pH value, secreting bactericidal proteins and stimulating epithelial cells to produce defensins (14). They can block the attachment or invasion of pathogens to epithelial cells by competing for surface receptors, enhance the intestinal barrier, promote nutrient absorption, regulate the immune system, and are widely applied in the fields of food, health supplements and medicine (15). Due to the functional differences among various types of *bifidobacteria*, the current research may not have fully exploited the full advantages of infant-derived *bifidobacteria*. Select the strains with excellent *bifidobacteria*, and study their inhibitory effect on *E. coli*. This is of great significance for improving the microecological balance of the human intestinal tract and enhancing health levels.

## 2 Materials and methods

### 2.1 Isolation and identification of Bifidobacteria

This study collected fecal samples from healthy infants aged 0–6 months in the Harbin area (*n* = 2). The inclusion criteria were: (1) Exclusive breastfeeding; (2) No history of any diseases; (3) No use of antibiotics. In this study, all sample collections were conducted with the written informed consent of the guardians/parents. According to the method of Ma et al. (16) and with some minor modifications. Fecal samples (2 g) were transferred into a fecal sampling device containing a prereduction solution, and were then placed in an ice box. The samples were serially diluted and cultured on a *Bifidobacterium* agar plate. All isolates were further purified by streaking on the agar plate and preliminarily identified based on their morphological characteristics and staining features (Gram-positive bacilli). The 16S rRNA gene sequences were amplified using the universal primers shown in Supplementary Table S1 and sequenced at Sangon Biotech Co., Ltd. (Shanghai, China). The sequences were analyzed by BLAST to identify the species. Infant feces samples were obtained with approval from the Human Research and Ethics Committee of Hospital in Northeast Agricultural University. The approval number is NEAUHOS20200013 and the study complies with the Helsinki Declaration.

### 2.2 Bacterial strains and cell cultures

All the strains were stored at −80 °C in 40% glycerol stock solution. *E. coli* ATCC25922 and *S. aureus* ATCC25923 were activated in LB medium at 37 °C for three generations.

The *bifidobacterium* strains were activated in the mMRS medium supplemented with L-cysteine chloride, and incubated anaerobically at 37 °C in an atmosphere of nitrogen. All the strains were passaged three times before the experiment.

The HT-29 cells were cultured in DMEM-High glucose medium and routinely in a 37 °C, 5% CO_2_ incubator. After the cells reached confluency, they were used in all downstream experimental analyses.

### 2.3 Sample preparation

#### 2.3.1 Preparation of indicator bacterial suspension

*E. coli* ATCC 25922 and S. aureus ATCC 25923 were harvested by centrifugation (8,000 rpm, 10 min, 4 °C), washed three times with sterile PBS, and resuspended in PBS. The bacterial concentration was adjusted to 1 × 108 CFU/mL for subsequent experiments.

#### 2.3.2 Preparation of Bifidobacterium cell-free supernatant (CFS)

Second-generation *bifidobacteria* were inoculated (2% v/v) into liquid medium and cultured anaerobically for 18 h. The culture was centrifuged (12,000 × g, 8 min, 4 °C), and the supernatant was sterile-filtered (0.22 μm) and stored at 4 °C.

### 2.4 The antibacterial activity of the isolated bifidobacteria

The Oxford cup method was used to evaluate antibacterial activity (17). Indicator bacterial suspensions were spread evenly on agar plates, followed by placement of sterile Oxford cups. Then, 100 μL of cell-free supernatant was added to each cup. After incubation (37 °C, 24 h), inhibition zone diameters were measured, and samples exhibiting clear zones with the largest diameters were selected for further analysis.

### 2.5 The probiotic characteristics of Bifidobacterium

#### 2.5.1 Simulated gastrointestinal digestion

The tolerance of *B. longum* to simulated gastrointestinal fluids was evaluated as described by Zhang et al. with modifications (18). Simulated gastric fluid (SGF) contained 3 mg/mL pepsin in PBS (pH 3.0, adjusted with 1 M HCl), while simulated intestinal fluid (SIF) consisted of 1 mg/ml trypsin and 0.3% bile salts in PBS (pH 8.0, adjusted with 1 M NaOH). All the fluids need to be filtered through a 0.22 μm filter membrane. bacterial suspension was added in SGF and incubated anaerobically for 3 h, followed by 3 h in SIF. Viable counts were determined by plating serial dilutions on mMRS agar, and survival rates were calculated.

Survival rate (%) = logS1/logS0 × 100

S0 is the number of colonies before treatment, and S1 is the number of colonies after treatment.

#### 2.5.2 Bacteriolytic enzyme resistance test

Following Lemos Junior et al. with modifications (19), *B. longum* D2, D1, D29, and D32 were inoculated into mMRS broth containing 100 mg/L lysozyme and incubated anaerobically (37 °C, 2 h). Viable counts were determined by plating serial dilutions on mMRS agar and counting colonies after 48 h anaerobic incubation.

#### 2.5.3 . Auto-aggregation and hydrophobicity

The auto-aggregation capacity of D2, D1, D29, and D32 was evaluated as described by Kang et al. (20). Bacterial suspensions were centrifuged (6,000 × g, 10 min), washed twice with PBS, and adjusted to OD_600_ = 0.6. After static incubation (1, 4, or 24 h), the absorbance of the supernatant was measured. Auto-aggregation percentage was calculated as:

Auto-aggregation (%) = 1 – A1/A0 × 100

A0 denotes the initial absorbance value, and A1 denotes the absorbance value after standing.

Bacterial hydrophobicity was determined by the xylene adhesion method (21). D2, D1, D29, and D32 were adjusted to OD_600_ 0.8–1.0 in PBS, mixed with xylene (1:3 v/v), and vortexed for 2 min. The aqueous phase was collected and incubated at 37 °C for 1 h, and then measured at OD_600_. The calculation of the hydrophobicity is as follows:

Cell surface hydrophobicity (%) = (H0–H1)/H0 × 100

H0 denotes the initial absorbance value, and H1 denotes the absorbance value of the lower aqueous phase after xylene treatment.

#### 2.5.4 Determination of antibiotic resistance

The antibiotic resistance profiles of D2, D1, D29, and D32 were evaluated using the disk diffusion method. Eight antibiotics were tested. Bacterial suspensions (100 μL) were spread on mMRS agar, and antibiotic disks were placed on the inoculated plates. After anaerobic incubation (37 °C, 48 h), inhibition zone diameters were measured and interpreted according to standard breakpoints (Supplementary Table S2).

#### 2.5.5 Comprehensive ability analysis

The four *Bifidobacterium* were ranked and scored from 4 points for highest performance to 1 point for lowest performance across each probiotic characteristic. Cumulative scores were calculated to determine overall probiotic potential.

### 2.6 Study on the antibacterial substances and activity of the target strain

#### 2.6.1 Analysis of antibacterial active ingredients

The antibacterial effects of CFS after three different treatments were evaluated: (1) Neutralization to pH 7.0 with 3 M NaOH; (2) Adjust the pH of CFS to 7, add Catalase at a final concentration of 5 mg/mL, and incubate at 37 °C for 2 h. Then restore its initial pH. (3) Protease K treatment with pH adjustment to 8.0. Add enzyme at a final concentration of 5 mg/ml and incubate at 37 °C for 2 h. Then restore its initial pH. 200 μL of treated or untreated (control) CFS was added to Oxford cups on *E. coli* ATCC 25922-inoculated LB agar plates. After 24 h incubation at 37 °C, inhibition zone diameters were measured.

#### 2.6.2 Determination of short-chain fatty acids

The content of short-chain fatty acids (SCFA) in CFS was determined by gas chromatography-mass spectrometry. Chromatographic column: HP: NNOWAX (30 m, 0.25 mm, 0.25 μm); injection port temperature: 220 °C; temperature ramping program: Maintain 60 °C for 1 min, then increase at a rate of 30 °C/min to 210 °C, and maintain for 3 min; ion source: EI source; ion source temperature: 230 °C; interface temperature: 230 °C.

#### 2.6.3 The influence of temperature and pH on the antibacterial effect

The CFS of D2 were respectively subjected to water bath at 40, 60, 80, and 100 °C for 1 h, and at 121 °C for 20 min. Subsequently, the inhibitory effects of the CFS on *E. coli* under different temperature conditions were investigated using the Oxford cup double-layer agar diffusion test. The untreated CFS was used as the control.

Using 1 mol/L NaOH, the pH of CFS was adjusted to different values of 2.0, 3.0, 4.0, 5.0, 6.0, 7.0 and 8.0. The influence of pH on the antibacterial effect of the CFS was investigated according to the above method.

#### 2.6.4 Determination of sub-inhibitory concentration (SIC) and minimum inhibitory concentration (MIC)

The method of Diao et al. (22) was referred to and slightly modified. The MIC and SIC values of the CFS for *E. coli* were determined using the two-fold dilution method. The *E. coli* was cultured to the logarithmic growth phase and OD_600_ was adjusted to 0.4. The CFS was diluted two-fold consecutively to 1,280, 640, 320, 160, 80, 40, 20, 10, 5 μL/mL. Add the adjusted CFS and *E. coli* suspension to a 96-well plate and incubate at 37 °C for 24 h. No visible growth at the lowest concentration of CFS was defined as its MIC, and no effect on growth was defined as SIC.

#### 2.6.5 . Growth curve and time-kill curve

*E. coli* (106 CFU/mL) was cultured with CFS at MIC or SIC, with OD600 measured every 2 h to generate growth curves. Samples collected at 4 h intervals (0–24 h) were serially diluted, plated on LB agar to construct time-kill curves, using untreated cultures as controls.

### 2.7 Study on the bactericidal mechanism

#### 2.7.1 Growth curve and time-kill curve

Following modified methods of Wang et al. (6), *E. coli* (106 CFU/mL in PBS) was treated with CFS at MIC or SIC for 3 h at 37 °C, while an equal volume of mMRS medium served as the blank control. Then examined for morphological alterations by Scanning Electron Microscope (SEM).

#### 2.7.2 . The influence of CFS on the leakage of nucleic acid and proteins

Based on the method of Diao et al. (22) with modifications, *E. coli* cell membrane integrity was assessed by quantifying released intracellular components in the supernatant. Bacterial cultures were grown in a shaking incubator at 37 °C (160–180 rpm) for 3 h. Aliquots were collected at 0, 30, 60, 90, 120, 150, and 180 min. Culture supernatants from the control, SIC, and MIC groups were filtered (0.22 μm) and analyzed for absorbance at 260 nm (nucleic acids) and 280 nm (proteins).

#### 2.7.3 Determination of ion permeability of cell membranes by conductivity

The method developed by Li et al. was modified for this study (23). *E. coli* was cocultured with CFS in a shaking incubator (37 °C, 180 rpm) for 6 h. At hourly intervals, aliquots were collected from each group, centrifuged ( × 10,000 g, 10 min, 4 °C), and the electrical conductivity of the resulting supernatants was measured. A parallel culture without CFS supplementation served as the control group.

#### 2.7.4 Assessment of cell wall integrity using Alkaline Phosphatase (ALP) assay

*E. coli* cells were resuspended in sterile PBS to an OD_600_ of 0.4. CFS was added to achieve final concentrations corresponding to the SIC and MIC. Cultures were incubated at 37 °C with shaking (160 rpm), and extracellular ALP activity was monitored at 0, 2, 4, 6, 8, and 10 h. At each time point, aliquots were centrifuged (6,000 × g, 8 min, 4 °C), and the supernatants were collected for ALP activity measurement using a commercial ALP detection kit according to the manufacturer's instructions.

#### 2.7.5 The inhibitory effect of CFS on biofilms

The anti-biofilm activity of CFS was evaluated using a crystal violet staining method based on Haney et al. and with modifications (24). Bacterial cultures (50 μL) were combined with 50 μL CFS in LB broth to achieve final concentrations corresponding to the MIC and SIC, then transferred to a 96-well plate. After 24 h incubation at 37 °C, untreated cells were used as the control. After the biofilm formation, the cell suspension was removed. The plate was washed twice with 150 μL PBS and dried at 37 °C for 20 min. Subsequently, 1% crystal violet was added to each well and the biofilm-forming cells were stained for 30 min. The plate was then rinsed and dissolved in a solution of 30% methanol and 10% acetic acid. The absorbance of each sample was measured at 600 nm using a microplate reader. Methanol and mMRS were selected as the positive control and the blank control respectively.

### 2.8 The research of CFS on the infection of HT-29 cells by E coli

#### 2.8.1 The effect of CFS on the adhesion of E. coli to HT-29 cells

Modified from Jacobsen et al. (25), competition, protection, displacement assay were conducted: (1) Competition: simultaneous incubation of HT-29 monolayers with 1 mL CFS (MIC/SIC) and 1 mL *E. coli* for 1 h; (2) Protection: pretreatment with CFS for 1 h before bacterial challenge; (3) Displacement: 1 h bacterial adhesion followed by CFS treatment. After washing, infected cells were lysed (0.5% Triton X-100, 8 min), serially diluted, and plated on LB agar for viable counts (37 °C, 48 h). The control group was only added with the E. coli suspension.

Inhibition rate of adhesion (%) = (N0–N1)/N0 × 100

N1 represents the number of *E. coli* ATCC25922 colonies after CFS treatment, and N0 represents the number of colonies without CFS treatment.

#### 2.8.2 Effect of CFS on E. coli invasion of HT-29 Cells

The experimental method is the same as 2.8.1. The cell culture medium containing gentamicin (100 mg/mL) is added to the cell well plate and incubated with the cells for 1 h to kill all extracellular bacteria. Then the cells are washed three times with PBS and lysed with 0.5% Triton X-100 for 8 min. The cell colony count is performed as described above.

Inhibition rate of invasion (%) = (N0–N2)/N0 × 100

N2 represents the number of *E. coli* colonies after CFS treatment, and N0 represents the number of colonies without CFS treatment.

### 2.9 An *E. coli*-induced enteric disorder mouse model

Fifty specific pathogen-free (SPF) male BALB/c mice, aged 6 weeks, were randomly allocated into five groups (*n* = 10 per group) as follows: the blank control group (NC), the model control group (MC), the positive control group treated with gentamicin sulfate (PC), and two experimental groups administered *Bifidobacterium* longum D2 and A14 (It has similar growth performance and probiotic properties compared with D2 but shows no antibacterial effect *in vitro*), respectively. Following a one-week acclimatization period, an intestinal disorder model was established from days 8 to 14 by daily intragastric administration of 0.2 mL of an *E. coli* suspension (1 × 108 CFU) to all groups except the NC group, which received an equal volume of phosphate-buffered saline (PBS). Subsequently, during the treatment period from days 15 to 28, the PC, D2, and A14 groups received daily intragastric doses of 0.2 mL of gentamicin sulfate (0.2 mg), D2 (1 × 10^9^ CFU), and A14 (1 × 10^9^ CFU), respectively. The NC and MC groups continued to receive PBS. Upon completion of the experiment, all mice were euthanized (Euthanize mice by cervical dislocation following anesthesia with 4%−5% isoflurane), and samples of feces, blood, and colon tissue were collected for subsequent analysis. all mice received humane care, and all experiments were reviewed and approved by the Northeast Agricultural University animal care and welfare committee under the approved protocol number NEAUEC 20240493.

### 2.10 Histopathology analysis

Histological analysis was performed on colon tissue samples. Briefly, the tissues were fixed by immersion in 4% neutral buffered formaldehyde, subsequently dehydrated, and embedded in paraffin. The embedded tissues were sectioned, mounted on glass slides, and stained with hematoxylin and eosin (H&E). The stained sections were then examined under a light microscope (Olympus BX53, Japan) at 40 × magnification for morphological assessment.

### 2.11 Analysis of cytokines in colon tissue and serum

The concentrations of typical pro-inflammatory factors (IL-1β, IL-6, IL-8, TNF-α) and the anti-inflammatory factor IL-10 were measured using commercial enzyme-linked immunosorbent assay (ELISA) kits, strictly in accordance with the manufacturer's instructions (Solarbio, China).

### 2.12 Assessment of intestinal barrier function and intestinal permeability

The mRNA expressions of intestinal barrier-related proteins (ZO-1, Occludin-1 and MUC2) were analyzed by RT-qPCR technology. The total RNA of the colon was obtained using the RNAiso Plus kit (Vazyme, China). First-strand cDNA was synthesized from total RNA using the Transcriptor First Strand cDNA Synthesis Kit (Promega, USA). Gene expression was quantified by real-time PCR using the Stormstar SybrGreen qPCR Master Mix (Promega, USA) on a Go Taq^®^ SYBR-Green qPCR Master Mix instrument. The following primer sequences were employed:

*ZO-1*(5′-3′: AACCCGAAACTGATGCTGTGGATAG, 3′-5′: CGCCCTTGGAA TGTATGTGGAGAG), *Occludin* (5′-3′: TTGGCTACGGAGGTGGCTATGG, 3′-5′: CCTTTGGCTGCTCTTGGGTCTG), and *MUC2* (5′-3′: CGAGCACATCACCTAC CACATCATC, 3′-5′: TCCAGAATCCAGCCAGCCAGTC).

The serum concentrations of lipopolysaccharide (LPS), D-lactate (D-LA), and diamine oxidase (DAO) were measured with ELISA kits (Solarbio, China) to evaluate intestinal permeabilit.

### 2.13 Statistical analysis

The results were shown as mean values ± standard error, and an analysis of variance (ANOVA) with Tukey's multiple comparisons was performed to analyze data under a significance level (*P* < 0.05) using a Statistix 8.0 software (Analytical Software, St. paul, MN, USA). Graphs were plotted using an GraphPad Prism 9.5 (Software, USA).

## 3 Results and discussion

### 3.1 Isolation and identification of Bifidobacterium strains

Fecal samples were collected from two healthy infants (0–6 months old) for *bifidobacterial* isolation. Primary isolation was performed on mMRS agar plates under anaerobic conditions (37 °C, 48 h). *Bifidobacterium* colonies were selected based on distinctive morphology: translucent to opaque white/milky-white coloration, moist convex circular surfaces with smooth edges (2–4 mm diameter) (Supplementary Figure S1a). Gram-positive staining and SEM image confirmed typical *bifidobacterial* cellular morphology, exhibit-ing Y-shaped, V-shaped, or blunt-ended rod structures (26) (Supplementary Figures S1b, c). Select the colonies that meet the above requirements for pure culture and identify them through 16S rRNA sequence analysis. The phylogenetic tree was constructed by comparing and analyzing using the BLAST program provided by the National Center for Biotechnology Information (NCBI), as shown in Supplementary Figure S1d. Phylogenetic analysis confirmed that all 38 presumptive *Bifidobacterium* isolates belonged to the *Bifidobacterium* genus, with the following distribution: 24 strains were identified as *Bifidobacterium longum* (*B. longum*) subsp. suis. 14 strains were classified as *Bifidobacterium bifidum* (*B. bifidum*).

### 3.2 Antibacterial activity of Bifidobacterium isolates

The antibacterial activity of probiotics is regarded as a key characteristic that limits the proliferation of pathogenic bacteria, thereby maintaining a healthy microbial balance in the intestinal tract (27). *Bifidobacteria* can inhibit the growth of pathogens by producing bioactive molecules such as organic acids, hydrogen peroxide, and bacteriocins (28). Among the 38 tested *Bifidobacterium* strains (Table 1), 26 strains exhibited varying degrees of antibacterial activity, with strain-dependent differences observed, consistent with prior findings (29). Notably, strains D2, D1, D29, and D32 exhibited antibacterial activity against both indicator strains (*E. coli* ATCC25922 and *S. aureus* ATCC25923). The inhibition zones against *E. coli* measured 20.72 mm (D2), 20.42mm (D29), 20.08 mm (D32), and 14.34 mm (D1), while those against S. aureus ranged from 8.24 mm (D32) to 9 mm (D2 and D1). As shown in Table 1, strains D2, D1, D29, and D32 exhibited inhibitory effects against both Gram-positive and Gram-negative pathogens, suggesting broad-spectrum antibacterial potential. Four strains exhibiting strong antibacterial activity were selected for subsequent experiments. Further investigation is needed to elucidate their antibacterial compounds and underlying mechanisms.

**Table 1:** Screening results of antibacterial *Bifidobacteria*.

Strain name	*E. coli* ATCC25922 diameter/mm	*S. aureus* ATCC25923 diameter/mm	Number of inhibiting bacteria
A1	+ (8.42 ± 0.04)	–(0)	1/2
A2	++ (13.02 ± 0.14)	–(0)	1/2
A6	-(0)	–(0)	0/2
A7	+ (8.21 ± 0.07)	–(0)	1/2
A9	+ (8.24 ± 0.05)	–(0)	1/2
A11	+ (8.35 ± 0.21)	–(0)	1/2
A14	-(0)	–(0)	0/2
A15	+ (8.43 + 0.02)	–(0)	1/2
A12	+ (8.31 ± 0.06)	–(0)	1/2
A5	+ (8.40 ± 0. 11)	–(0)	1/2
A18	+ (8.36 ± 0.21)	–(0)	1/2
B4	++ (13.52 ± 0. 16)	–(0)	1/2
D15	+ (8.32 ± 0.03)	–(0)	2/2
D37	+ (8.41 ± 0.05)	–(0)	1/2
D2	+++(20.72 ± 0.39)	+ (9 ± 0.02)	2/2
D21	++ (14.02 ± 0.21)	–(0)	1/2
D1	++ (14.34 ± 0.05)	+ (9 ± 0.34)	2/2
D3	–(0)	–(0)	0/2
D5	–(0)	–(0)	0/2
D6	–(0)	–(0)	0/2
D7	–(0)	–(0)	0/2
D10	+ (8. 12 ± 0.24)	–(0)	1/2
D11	++ (12.04 ± 0. 12)	–(0)	1/2
D12	+ (8.04 ± 0.05)	–(0)	1/2
D13	–(0)	–(0)	0/2
D17	++ (14.03 ± 0.02)	–(0)	1/2
D20	–(0)	–(0)	0/2
D22	–(0)	–(0)	0/2
D23	+ (9.31 ± 0.21)	–(0)	1/2
D24	+ (9.42 ± 0. 14)	–(0)	1/2
D28	++(14.25 ± 0.02)	–(0)	1/2
D29	+++ (20.42 ± 0.05)	+ (8.45 ± 0.01)	2/2
D32	+++ (20.08 ± 0.63)	+ (8.24 ± 0.03)	2/2
D33	+ (9.32 ± 0.05)	–(0)	1/2
D34	–(0)	–(0)	0/2
D38	+ (8.21 ± 0.05)	–(0)	1/2
D41	–(0)	–(0)	0/2
D42	–(0)	–(0)	0/2

The diameter of the Oxford cup is 8 mm; ‘–' indicates no inhibition zone/diameter ≤ 8 mm, ‘+' indicates a diameter of 8–10 mm, ‘++'indicates a diameter of 10–15 mm, and ‘+++' indicates a diameter >15 mm.

### 3.3 The probiotic properties of Bifidobacterium strains

During gastrointestinal transit, probiotic face significant challenges, such as the low pH conditions in the stomach, bile salts, and digestive enzymes in the intestines (30). To fully utilize their beneficial effects on the host, it is important to consume a sufficient amount of live bacteria. The International Dairy Federation recommends at least 106 CFU/g (31). Among the tested strains, as shown in Supplementary Figure S2a: in the simulated gastric juice for 3 h, the survival rates of strains D2, D1, D29, and D32 were 85.21%, 82.52%, 83.79%, and 69.54% respectively. In the simulated intestinal juice for 3 h, the survival rates of the strains were 73.38%, 60.32%, 70.40%, and 50.48% respectively. Supplementary Figure S2b demonstrates that all tested strains maintaining viability exceeding 70% following lysozyme exposure. Strain D2 exhibited the highest survival rate (85.79%), followed by D1 (82.06%), D29 (78.15%), and D32 (70.08%). The results demonstrate that strain D2 exhibited significantly greater digestive resistance compared to other tested strains. However, D32 showed the lowest tolerance (*P* < 0.05).

Bacterial self-aggregation facilitates intestinal persistence through enhanced cell-cell adhesion and aggregate formation (32). Cell surface hydrophobicity represents one of the key mechanisms mediating bacterial adhesion to intestinal epithelial cells (33). As shown in Supplementary Figure S2c: The self-aggregation rates were all above 70% after 24 h of incubation. The self-aggregation rates of each strain were in the following order: 87.41% (D2) > 81.82% (D1) > 73.35% (D29) > 71.22% (D32). The results of the hydrophobicity of the four strains of *Bifidobacterium* are shown in Supplementary Figure S2d. The order of hydrophobicity is D2 (36.42%) > D29 (27.15%) > D1 (19.35%) > D32 (15.01%). The results indicate that D2 is considered to have a relatively strong potential for adhering to epithelial cells. The antibiotic susceptibility of four *Bifidobacterium* was evaluated against eight antibiotics. *E. coli* served as the quality control strain, with results interpreted according to CLSI performance standards. Supplementary Table S3 summarizes the antibiotic susceptibility profiles of the four *Bifidobacterium*, revealing they all have varying degrees of sensitivity to antibiotics. D2 was consequently selected for subsequent comparative analyses based on comprehensive evaluation criteria (Supplementary Figure S2e).

### 3.4 Research on antibacterial substances in CFS

Probiotics suppress *E. coli* growth through multiple antimicrobial mechanisms, including organic acid production (e.g., lactic and acetic acids), hydrogen peroxide secretion, and bacteriocin synthesis (34, 35). The antibacterial activity of *Bifidobacterium* CFS against *E. coli* ATCC25922 after different treatments was determined, as shown in Supplementary Table S4. The CFS of D2 and were adjusted to pH = 7.0. The Antimicrobial activity of D2 against *E. coli* was significantly lower than that of the control group (*P* < 0.05). The diameter of the inhibition zone of D2 decreased by 35.7%. After treatment with hydrogen peroxidase, the inhibition zone diameters of CFS were not significantly different from those of the control group (*P* > 0.05), indicating that D2 did not produce hydrogen peroxide in CFS, or the amount of hydrogen peroxide produced was insufficient to inhibit the growth of *E. coli*. Proteinase K treatment significantly reduced the antimicrobial activity of CFS against *E. coli*, with the diameters of the inhibition zones for D2 decreased by 16.7% compared to untreated controls (*P* < 0.05). This proteolysis-sensitive inhibition suggests the presence of proteinaceous antimicrobial compounds, such as bacteriocins or antimicrobial peptides, in the CFS. In conclusion, after the three different treatments, adjusting the pH of CFS had the greatest impact on the antibacterial ability of D2. The organic acids might be the effective antibacterial substances.

**Figure 1:**
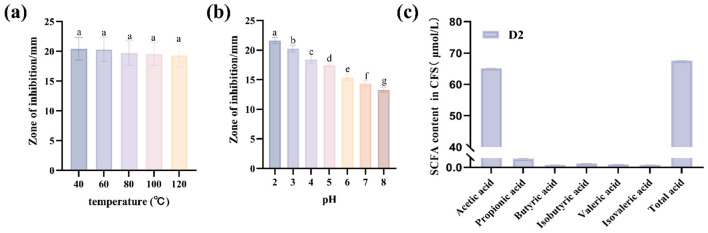
The antibacterial substances and activity of CFS of D2. **(a)** The antibacterial effect of CFS of D2 on *E. coli* at different temperatures. **(b)** The antibacterial effect of CFS of D2 on *E. coli* at different pH. **(c)** content of SCFA in CFS. Data are represented as mean ± SD (*n* = 3). Significant differences (*P* < 0.05) among different group are indicated by different letters.

The results of the inhibitory effects of CFS from D2 on *E. coli* at different temperatures are shown in Figure 1a. Under the conditions of 40 °C−121 °C, there was no significant difference in the inhibition zones of the CFS from D2 on *E. coli* (*P* > 0.05), and the relative inhibitory activities of strain were above 90%. Although treatment with proteinase K significantly reduced the antibacterial activity of the strain of CFS (*P* < 0.05), the activity did not show significant changes after heat treatment at 40 °C−120 °C. This seemingly contradictory phenomenon may be due to: (1) the bacteriocins in the strain of CFS exhibit thermal stability. Similarly, in the study by Lei et al. (36), part of their antibacterial activity remained after the bacteriocin were exposed to short-term high-temperature treatment. (2) Organic acids as the main antibacterial components, do not have their activity affected by temperature. The overall antibacterial efficacy is maintained through the synergistic effect between heat-resistant proteins and organic acids.

The antibacterial abilities of CFS after different pH treatments were evaluated, as shown in Figure 1b. The antibacterial effect of CFS of D2 on *E. coli* was affected by changes in pH. The inhibition zone diameters of CFS against *E. coli* decreased as the pH value increased. The inhibition zones of CFS produced by the D2 against *E. coli* showed significant differences at pH 2.0 compared with other pH (*P* < 0.05), and the inhibitory effect was the greatest. The results indicate that the inhibitory effects of the D2 may be related to pH changes. This is also consistent with the data in Supplementary Table S4. Ibrahim and Bezkorovainy demonstrated that the growth inhibition of *E. coli* by *Bifidobacterial* broths is primarily pH-dependent (37). The broths contain protonated organic acids (e.g., lactic and acetic acids) that can diffuse across the bacterial cytoplasmic membrane. Following intracellular entry, these acids dissociate, releasing protons that disrupt critical metabolic processes in *E. coli*. Therefore, the content of short-chain fatty acids in the CFS of D2 was measured and shown in Figure 1c. The acetic acid content in the CFS of D2 was the highest, at 65.14 μmol/L. *Bifidobacteria* degrade the hexose sugars glucose and fructose through a unique pathway named “bifid shunt” and mainly produce two short-chain fatty acids, namely acetic acid and lactic acid. Similar conclusions have also been reached by Bruna Higashi et al. The acidity and the content of organic acids are related to the inhibitory activity of the *Lactobacillus* strains (38). In conclusion, based on the antibacterial properties of CFS and the acetate levels, acetate is hypothesized to be the main antibacterial substance of CFS for D2.

### 3.5 Study on antibacterial activities against *E. coli*

As shown in Table 2, the growth of *E. coli* was completely inhibited when the concentration of CFS was ≥ 320 μL/mL. The MIC was determined to be 320 μL/mL. The bacterial growth was not significantly affected when the concentration was ≤ 80 μL/mL, so the SIC was defined as 80 μL/mL.

**Table 2:** Experimental determination of MIC and SIC in cell-free fermentation.

Strain/OD_600_	Potency (μL/ml)										
	1,280	640	320	160	80	40	20	10	5	2.5	Blank
D2	0.202	0.224	0.245	0.277	0.281	0.326	0.344	0.351	0.372	0.373	0.38

**Figure 2:**
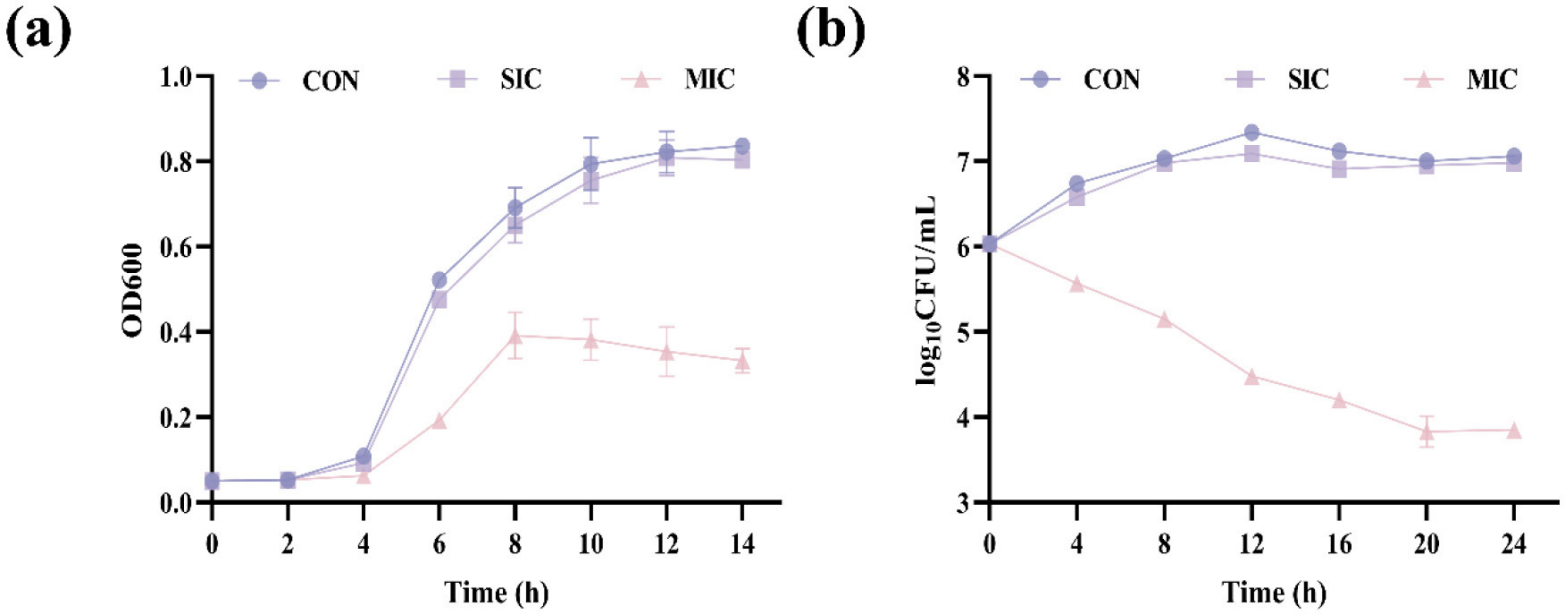
The antibacterial effect of CFS. **(a)** The effect of CFS of D2 on the growth performance of *E. coli*. **(b)** Time-kill curves of D2′s CFS against *E. coli*.

The influence of D2 on the growth performance of *E. coli* is shown in Figure 2a. In the SIC and control groups, the OD_600_ gradually increased from 0 to 14 h. The OD_600_ of the SIC groups were 0.803, while the control group was 0.836 at 14 h. The MIC groups of D2 showed no significant change in OD_600_ during the 0–4 h. There was an upward trend from 4 to 8 h. The OD_600_ of the MIC group was lower than that of the SIC group and the control group under the same time conditions. The OD_600_ were reduced to 0.333 after the *E coli* was treated with the 2′ MIC. The experiment demonstrated that the growth of *E coli* could be significantly inhibited by the CFS at MIC, while there was no obvious effect at SIC.

The time-kill curve of D2 against *E. coli* is presented in Figure 2b. During the 0–12 h period, the colony counts of *E. coli* in both the SIC treatment groups and the control groups showed an increasing trend and reached their maximum values at 12 h. The colony counts all tended to stabilize after 12 h, and the changing trends were basically consistent. Importantly, *E. coli* showed a rapid decline trend within 0–12 h after being treated with the MIC of D2. The colony count was reduced to its lowest value, which was 3.85 Log10CFU/mL at 24 h. The results show that after the SIC of CFS from D2 was applied to *E. coli*, the survival of the bacteria was almost unaffected. The CFS at MIC exhibited antibacterial and bactericidal effects on *E. coli*, which is consistent with the results of the CFS's impact on the growth curve.

### 3.6 Changes in cell morphology of *E. coli*

**Figure 3:**
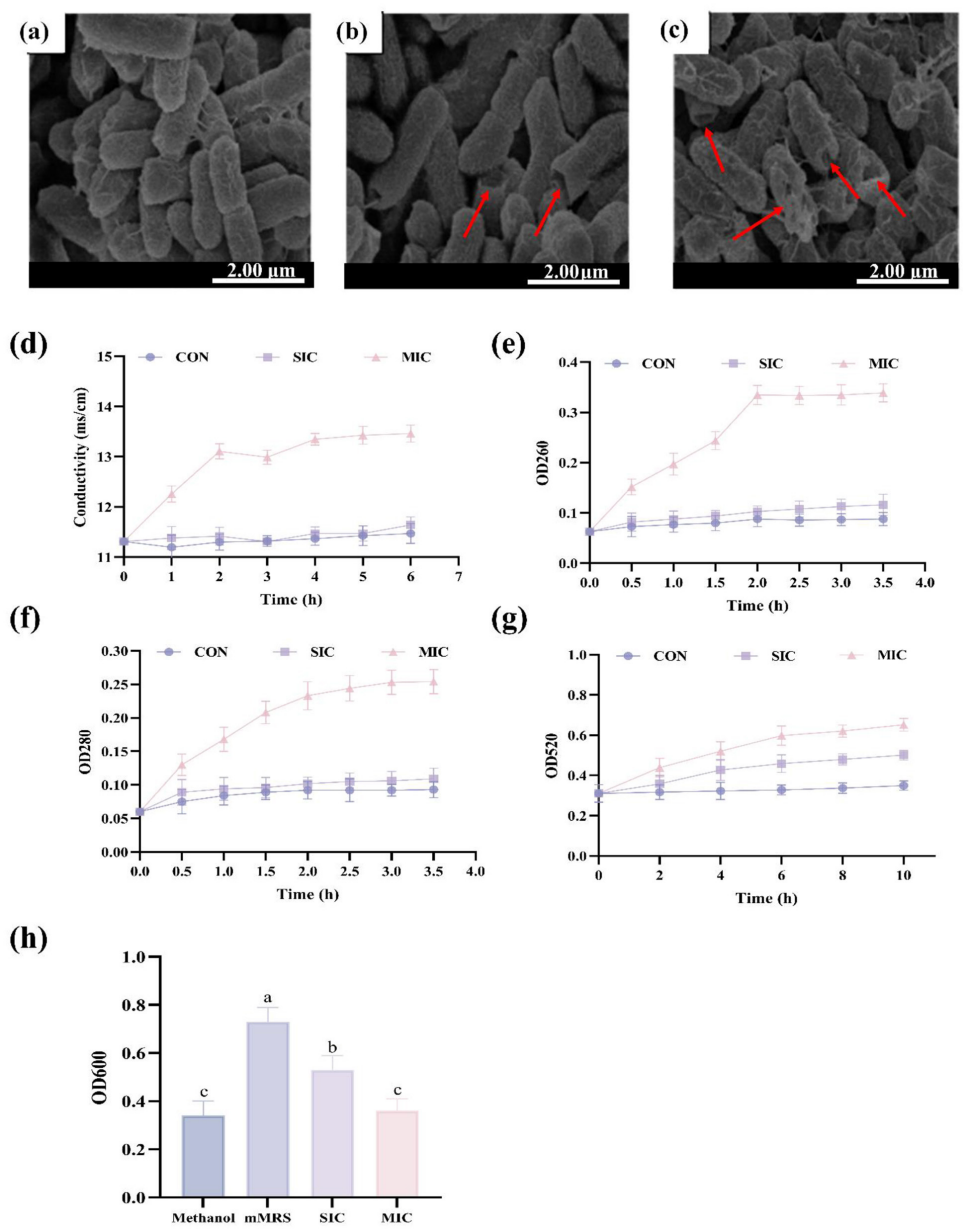
Investigation of antibacterial mechanisms. **(a–c)** SEM image of *E. coli* after CFS treatment. **(d)** Changes in electrical conductivity after treatment with D2′s CFS. **(e)** Nucleic acid leakage. **(f)** Protein leakage. **(g)** ALP release. **(h)** The effect of CFS on the formation of *E. coli* biofilms. Data are represented as mean ± SD (*n* = 3). Significant differences (*P* < 0.05) among different group are indicated by different letters.

Cell integrity is one of the bases for maintaining various physiological mechanisms of cells. The morphological changes of *E. coli* after being treated with CFS for 3 h are shown in Figure 3. The E. coli displayed characteristic rod-shaped morphology with uniform cell dimensions and an intact surface structure (Figure 3a). *E. coli* exhibited alterations, including irregular cell shapes, surface wrinkling, partial collapse, and enhanced cellular damage after being treated with SIC of D2 (Figure 3b). After the CFS treatment at the MIC of the D2, the cell damage caused was more severe. The surface of *E. coli* treated with the CFS of D2 at the MIC showed a large number of pores, cavities, depressions, and obvious aggregation and adhesion phenomena (Figure 3c). It can be inferred that the cavities formed due to the destruction of the cell membrane might be one of the reasons that inhibit the growth of *E coli*. Similarly, Wang et al. studied the morphology of the bacteria under SEM (39). The results showed that at the MIC, lactic acid could induce the disruption of the cell membrane integrity of *E. coli*. Antibacterial substances such as gallic acid can also inhibit the growth of *E. coli* by increasing the permeability of the membrane, inducing membrane leakage, and destroying its integrity (40). In this study, the SEM observation results indicated that the antibacterial activity of CFS was achieved through the membrane damage mechanism. The distortion of the cellular physical structure would lead to an increase in the instability of the cell membrane, thereby causing the leakage of intracellular contents (41). Therefore, it is necessary to conduct further research on the impact of CFS on the leakage of bacterial cell contents.

### 3.7 The impact on the integrity of the cell membrane

The cell membrane is regarded as an important structural component of bacteria and is also the main site of action for various antibacterial agents (42). Damage to cell membrane integrity leading to the leakage of intracellular contents. Thus, measuring the release of cellular components serves as a key indicator of membrane disruption, which can be quantitatively assessed through relative conductivity changes (43). At the SIC group and the control group, the conductivity increased slightly from 0 to 6 h as shown in Figure 3d. The conductivity of the SIC groups of D2 was 11.64 ms/cm at 6 h, while control group was 11.47 ms/cm. The conductivity of the MIC group increased with the passage of time. During the 0–2 h period, the conductivity rose rapidly. From 2 to 6 h, the conductivity of the strain showed a slow increasing trend and eventually stabilized at a certain value, which was 13.46 ms/cm.

When the cell membrane of *E. coli* is damaged, large molecules such as nucleic acids and proteins will leak out of the cell (44). The nucleic acid leakage caused by CFS in *E coli* is shown in Figure 3e: In the MIC group of D2, the OD_260_ increased rapidly over time, reaching a maximum of 0.335 within 2 h, and then remained relatively stable thereafter. After 3.5 h of treatment with 2′s CFS at MIC, the nucleic acid leakage of *E. coli* was 3.85 times that of the control group. Similarly, the leakage of intracellular proteins in *E. coli* was also evaluated. As shown in Figure 3f, after treatment with strains D2′ CFS at SIC, the protein concentration of *E. coli* did not show significant changes with the increase of time. The OD_280_ values were all between 0.09 and 0.1. The OD_280_ was significantly increased after MIC treatment. At 3.5 h, the OD_280_ of the MIC treatment group was 0.25.

The results demonstrate that CFS at MIC significantly alters bacterial membrane permeability, disrupting cellular osmotic balance and inducing substantial leakage of intracellular ions, as evidenced by increased system conductivity. More critically, CFS compromises membrane integrity at MIC, facilitating extracellular release of cytoplasmic nucleic acids and proteins.

### 3.8 Influence on cell wall integrity

The bacterial cell wall is an important structure that maintains the cell's osmotic pressure, shape and integrity, and is crucial for the vitality of the bacteria. The ALP in bacteria is mainly located in the periplasmic space. Therefore, the extracellular ALP activity can be used to assess the integrity of the bacterial cell wall. As shown in Figure 3g, the ALP enzyme activity of the control group's bacterial suspension remained at a relatively low level. The OD_520_ value was 0.35 at 10 h. After the CFS of D2 treatment, the ALP enzyme activity of the bacterial suspension gradually increased over time. The OD_520_ values of the bacterial suspension in the SIC group was 0.501, while those in the MIC group were 0.652, respectively. The CFS of the D2 have a destructive effect on the cell wall of *E. coli*. The degree of destruction at the MIC is higher than that at the SIC. The extracellular membrane and periplasmic space of *E. coli* will be severely damaged by higher concentrations of organic acids, resulting in increased permeability and leakage of ALP (45).

### 3.9 Inhibition of biofilm formation

The formation of biofilms is an important self-defense mechanism for bacteria (46). As shown in Figure 3h: In the blank control group with mMRS addition, the OD_600_ of *E. coli* suspension reached its maximum value, indicating the production of a large amount of biofilm. The biofilm was significantly reduced after adding CFS at the SIC (*P* < 0.05). The biofilm was further reduced when the CFS concentration increased to the MIC, and there was a significant difference compared with the SIC group (*P* < 0.05). Importantly, after treatment with D2, there was no significant difference between the MIC group and the methanol group (*P* > 0.05). These results indicate that the inhibitory ability of CFS on the biofilm of *E. coli* increases with the increase in concentration. Biofilms can be inhibited by short-chain fatty acids, and this is achieved through a quorum-sensing system mediated by competence-stimulating peptide (47).

### 3.10 Effects of CFS on adhesion and invasion of *E. coli* to HT-29 Cells

**Figure 4:**
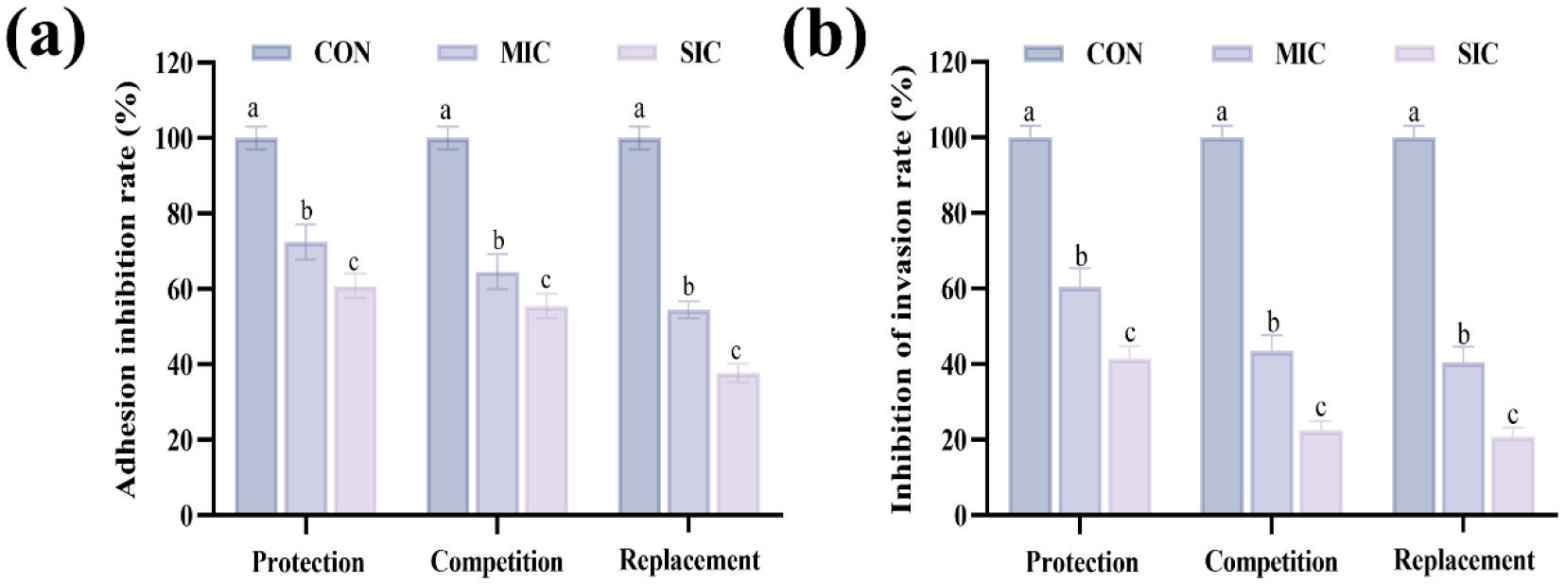
Effects of D2′s CFS on adhesion and invasion of *E. coli* to HT-29 Cells. **(a)** Inhibition rate of adhesion. **(b)** Inhibition rate of invasion. Data are represented as mean ± SD (*n* = 3). Significant differences (*P* < 0.05) among different group are indicated by different letters.

The HT-29 cell line isolated from colon adenocarcinoma has been widely employed to study the adhesion ability of probiotics (48). The probiotic strains can adhere to the GIT receptors and block the pathogens, thereby preventing adhesion (49). Based on the above research on the antibacterial mechanism, it was found that D2 had a strong ability to inhibit the cell membrane, cell wall, and the formation of biofilms of *E. coli*. Therefore, D2 was selected for the cell experiment. The CFS of D2 at SIC and MIC for inhibiting the adhesion of *E. coli* to HT-29 cells is shown in Figure 4a. The adhesion of *E coli* to HT-29 cells was 100% in the control group. Importantly, in the protection assay, *E. coli* adhesion was inhibited after SIC treatment, with an inhibition rate of 60.8%, and the inhibition rate was 72.44% at MIC. The inhibition rate significantly decreased compared with the SIC group (*P* < 0.05). Similarly, similar results were also obtained in the competition and displacement assay.

An important function of probiotics is to protect the host's intestinal tract from invasion by pathogens (50). The ability of D2 to inhibit the invasion of *E. coli* into HT-29 cells is shown in Figure 4b. In the protective assay, the MIC group's inhibition rate of invasion was 60.41%, which was significantly higher than that of the SIC group (41.42%) (*P* < 0.05). Similarly, in competitive and displacement assay, the inhibition rate of the MIC group was also significantly higher than that of the SIC group (*P* < 0.05). The results showed that 2′s CFS significantly inhibited the invasion of E. coli into HT-29 cells under MIC and SIC (*P* < 0.05) and there is a dose-dependent relationship. MIC showed good inhibitory activity against *E. coli* invasion of HT-29 cells.

### 3.11 Bifidobacteria alleviate intestinal damage and disorders caused by *E. coli*

**Figure 5:**
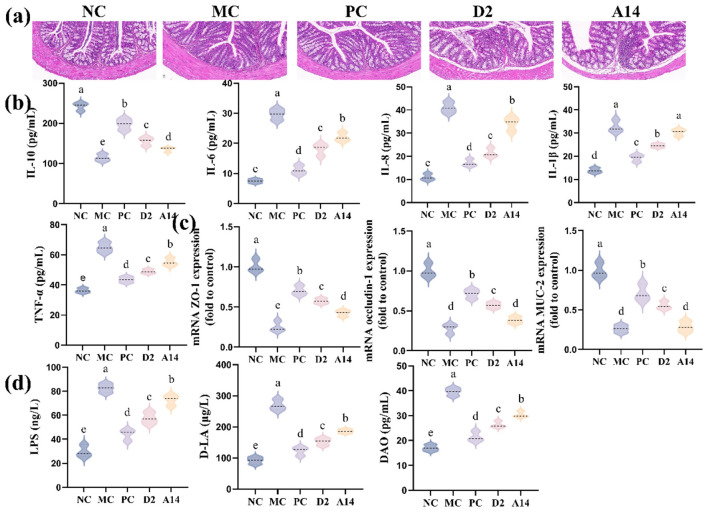
D2 alleviates the damage to the intestinal tract caused by *E. coli*. **(a)** Representative micrographs of colon tissue stained with H&E. **(b)** Levels of cytokines in colon tissue. **(c)** The mRNA expression level of tight junction proteins. **(d)** Indicators related to intestinal permeability. Data are represented as mean ± SD (*n* = 3). Significant differences (*P* < 0.05) among different group are indicated by different letters.

**Figure 6:**
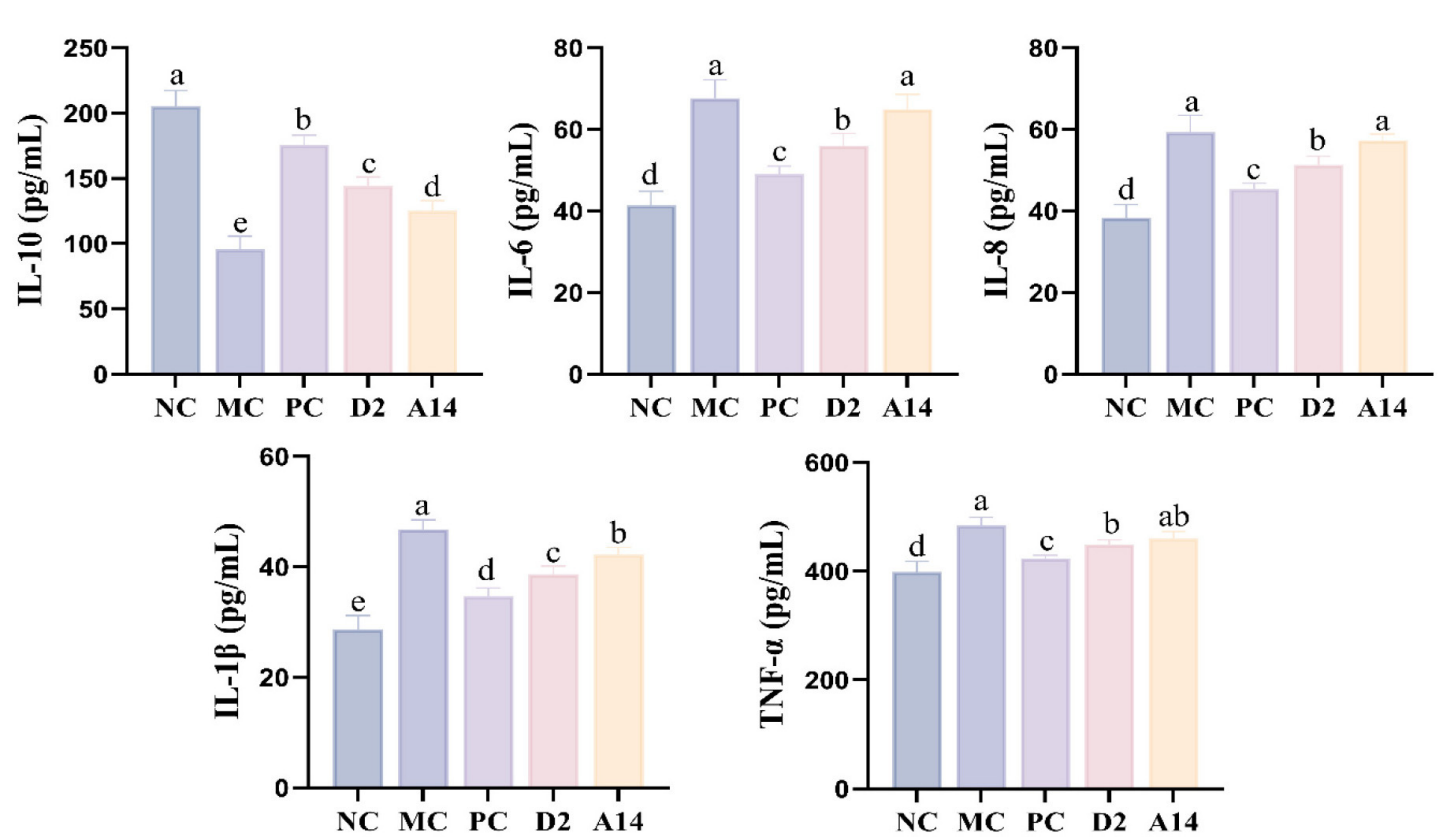
Cytokine levels in serum. Anti-inflammatory factor and proinflammatory factor levels. Data are represented as mean ± SD (*n* = 3). Significant differences (*P* < 0.05) among different group are indicated by different letters.

The intracellular Mitogen-activated protein kinases (MAPK) and Nuclear Factor-κB (NF-κB) signaling pathways can be activated by *E. coli* infection, thereby promotes the transcription of immune cells and leads to the release of a large amount of inflammatory factors (51). As shown in Figure 5a, histopathological analysis of colon tissue revealed that the MC group exhibited disrupted crypt architecture and substantial inflammatory cell infiltration. In contrast, the colonic morphology of the D2 group was similar to that of the NC, with intact epithelium and clear crypt structures, indicating that D2 can significantly promote the repair of the colonic tissue. At the cytokine level, D2 significantly increased the expression of the anti-inflammatory factor IL-10 and de-creased the levels of pro-inflammatory factors (IL-6, IL-8, IL-1β, and TNF-α) in colon tissue following *E. coli* infection (Figure 5b). Furthermore, D2 significantly increase the expression level of tight junction proteins (Figure 5c) and reduces the levels of LPS, D-LA, and DAO in the serum (Figure 5d) by restoring the function of the intestinal barrier. Based on its outstanding role in barrier repair, the serum inflammatory indicators were further evaluated. The results showed that *E. coli* infection led to a decrease in the level of serum anti-inflammatory factor, while the levels of pro-inflammatory factors IL-6, IL-8, IL-1β and TNF-α increased; both D2 and A14 treatments could reverse these changes, and the effect of D2 was better than that of A14 (Figure 6).

**Figure 7:**
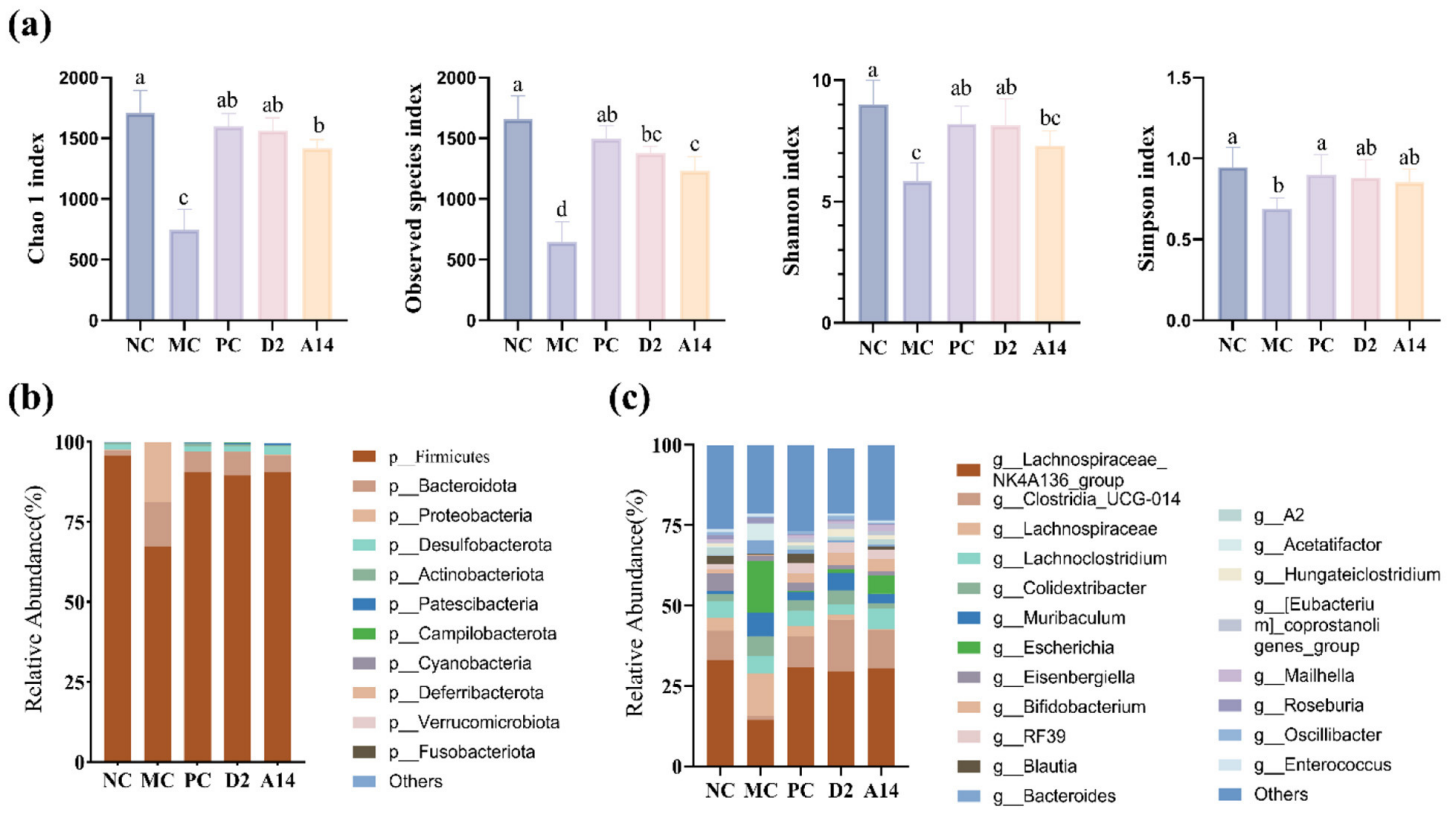
D2 regulates the intestinal microbiota of mice with intestinal disorders. **(a)** Analysis of gut microbiota α-diversity **(b)** The relative abundance of gut microbiota at the phylum level. **(c)** The relative abundance of gut microbiota at the genus level.

Probiotics can colonize in the intestines of animals, producing organic acids or other antibacterial substances to inhibit the growth of pathogens (52). the effect of D2 and A14 on the gut bacteria was estimated. D2 effectively ameliorated *E. coli*-induced gut microbiota dysbiosis and restored microbial diversity as shown in Figure 7a. At the phylum level (Figure 7b), the dominant bacterial phyla were Firmicutes and Bacteroidetes. Across all experimental groups, the average relative abundance of Firmicutes exceeded 85%. D2 significantly reversed the *E. coli*-induced reduction in the relative abundance of the Firmicutes (including many beneficial bacteria known for producing organic acids). Importantly, D2 significantly reduced the abundance of the Proteobacteria (which includes pathogens like *E. coli*), and its effect is superior to A14 which showed no antibacterial effect in the *in vitro* screening. At the genus level (Figure 7c), the proportion of Escherichia in the MC group was 15.99%. D2 significantly reduced the abundance of Escherichia (1.17%), and its effect was better than A14 (5.64%). Furthermore, the important bacteria that produce butyric acid, such as *Lachnospiraceae*_NK4A136_group and *Clostridia*_UCG-014, were significantly restored by D2, indicating that D2 also has the effect of promoting the abundance of beneficial gut bacteria.

## 4 Conclusion

This study first isolated 38 strains of *Bifidobacterium* from the feces of two healthy infants. D2 demonstrates the best antibacterial effect and probiotic properties. The main antibacterial substance in the CFS of D2 is acetic acid. CFS exerts its antibacterial effect by disrupting the integrity of the cell wall and cell membrane, leading to the leakage of cellular contents. Furthermore, CFS can weaken the adhesion and invasion of *E. coli* to HT-29 cells. The experiments *in vivo* demonstrated that D2 alleviated intestinal damage by reversing the expression of cytokines and intestinal junction proteins. D2 can reduce the abundance of *Escherichia* species and increase the abundance of beneficial species to regulate the intestinal flora disorder caused by *E. coli*. This suggests that D2 may be a potential probiotic strain for alleviating *E. coli*-induced intestinal microbiota dysbiosis.

## Data Availability

The original contributions presented in the study are included in the article/supplementary material, further inquiries can be directed to the corresponding authors.
