# Supplementary material for: Infant-derived Bifidobacterium strains screened in vitro for alleviating intestinal disorder caused by Escherichia coli
Source: Front Nutr. 2026 May 15;13:1788810. doi: 10.3389/fnut.2026.1788810 (PMC13219339; doi:10.3389/fnut.2026.1788810)
Supplement: Supplementary file 1 [file Table_1.docx]

Supplementary Material

# Supplementary Figures and Tables

## Supplementary Figures


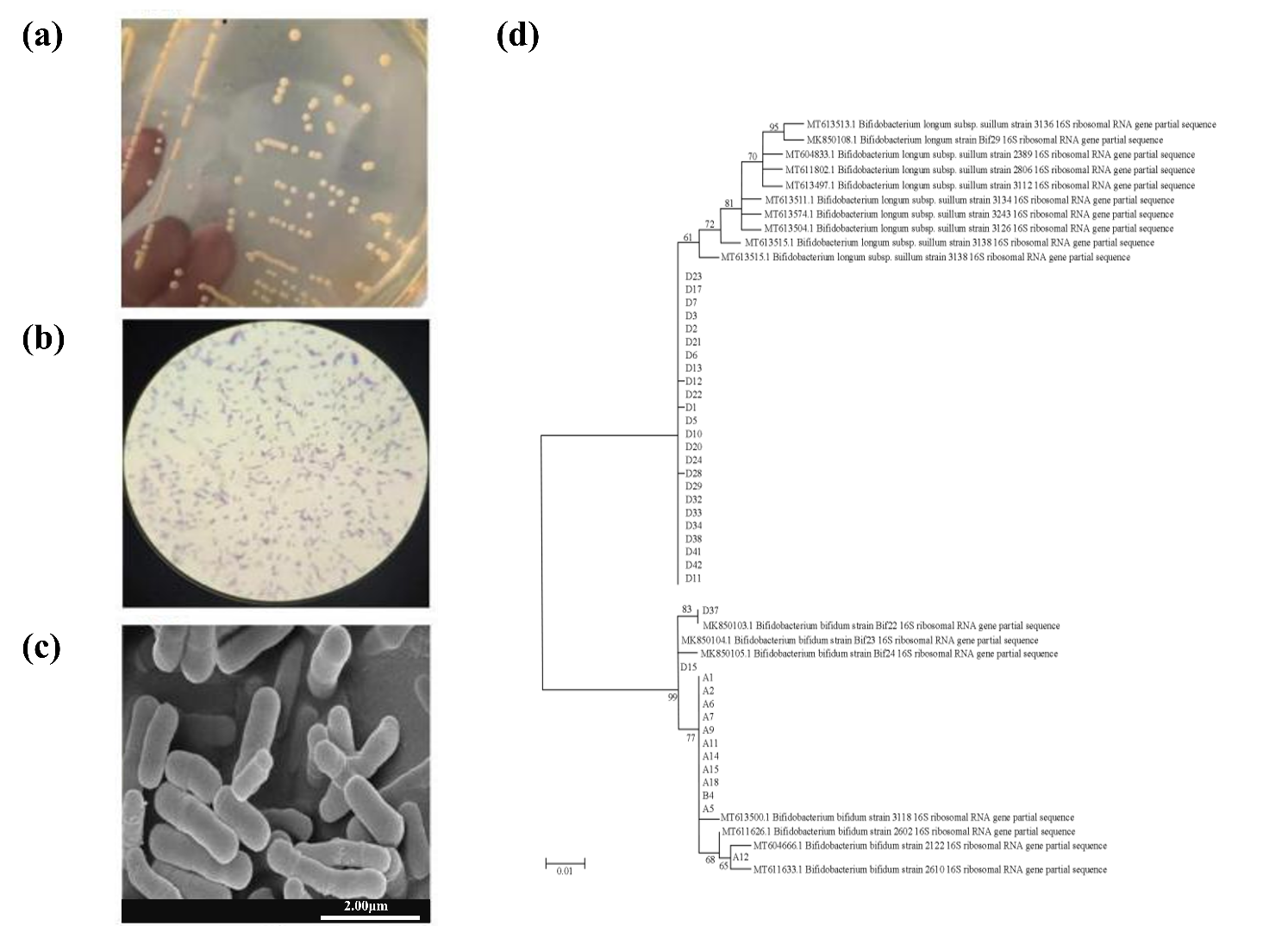


**Supplementary Figure 1.** Isolation and identification of *bifidobacteria*. (a) Colony morphology of *Bifidobacterium* isolate. (b) and (c) Cell morphology of *Bifidobacterium* isolate. (d) phylogenetic tree based on 16S rRNA gene sequences.


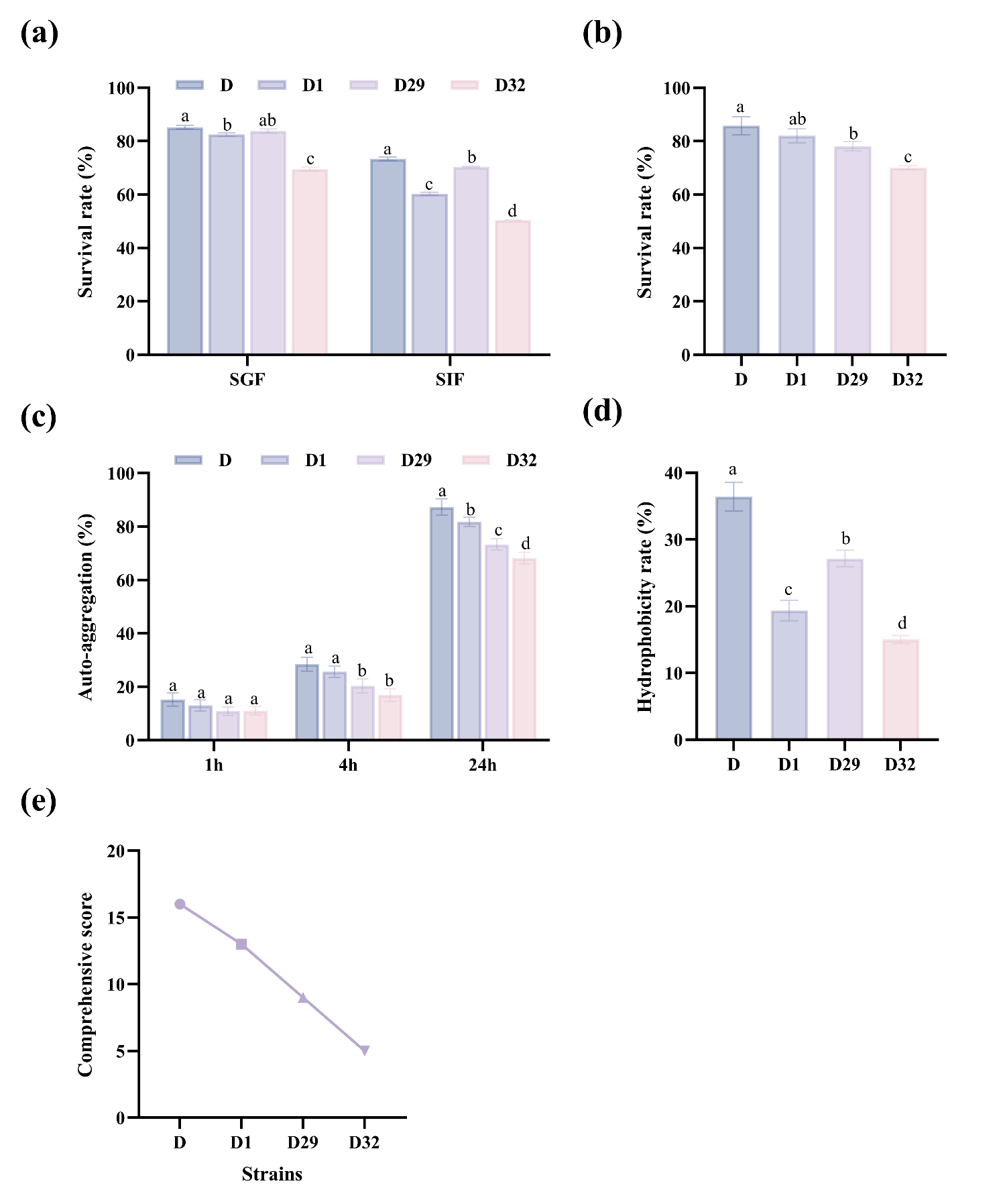


**Supplementary Figure 2.** Basic probiotic properties of *Bifidobacterium*. (a) Survival rate following simulated gastric digestion. (b) Survival rate following simulated intestinal digestion (c) Auto-aggregation. (d) Hydrophobicity. (e) Comprehensive Score. Data are represented as mean ± SD (n = 3). Significant differences (*P* < 0.05) among different group are indicated by different letters.

## Supplementary Tables

Table S1 Primers for 16S rRNA gene amplification.

| Gene name | Primers sequence (5’→3’) |
| --- | --- |
| 16S rRNA | 27 F: 5 ′-AGAGTTTGATCCTGGCTCAG-3 ′  1495 R:5 ′-CTACGGCTACCTTGTTACGA-3 ′ |

Table S2 Standards for determining antibiotic resistance

| Name | drug content (μg） | Judgement criteria（ mm) | | |
| --- | --- | --- | --- | --- |
|  |  | R | I | S |
| Penicillin G | 10 | ≤19 | 20-27 | ≥28 |
| Clindamycin | 2 | ≤14 | 15-20 | ≥21 |
| Ampicillin | 15 | ≤13 | 14-22 | ≥23 |
| Erythromycin | 30 | ≤14 | 15-18 | ≥19 |
| Rifampicin | 10 | ≤13 | 14-16 | ≥17 |
| Vancomycin | 5 | ≤16 | 17-19 | ≥20 |
| Gentamycin | 30 | - | - | ≥15 |
| Tetracycline | 10 | ≤12 | 13-14 | ≥15 |

Table S3 Screening results of antibacterial *Bifidobacteria*

| AST paper | Strains and diameter of inhibition zone in mm of the antibiotic tested | | | |
| --- | --- | --- | --- | --- |
|  | D29 | D1 | D32 | D2 |
| Penicillin G | 28.67±0.05 | 18.50±0.03 | 19.71±0.03 | 17.82±0.05 |
|  | S | R | R | R |
| Clindamycin | 8.83±0.02 | 8.30±0.06 | - | 19.88±0.03 |
|  | R | R | R | S |
| Ampicillin | 25.57±0.05 | 30.54±0.02 | 26.72±0.06 | 35.01±0. 10 |
|  | S | S | S | S |
| Erythromycin | 18.02±0.06 | 16.37±0.05 | 16.66±0. 15 | 9.03±0.06 |
|  | I | I | I | R |
| Rifampicin | 20.43±0.06 | 20.70±0. 12 | 20.45±0.05 | 21.52±0.05 |
|  | S | S | S | S |
| Vancomycin | - | - | - | 24.69±0. 18 |
|  | R | R | R | S |
| Gentamycin | 10.24±0. 14 | 10.32±0. 14 | - | 11.34±0.02 |
|  | R | R | R | R |
| Tetracycline | 17.22±0.54 | 14.36±0.26 | 11.23±0. 12 | 25.53±0.03 |
|  | I | R | R | S |

Note: The diameter of the inhibition zone is expressed as the mean ± standard deviation; ‘-’ indicates no inhibition zone; R indicates resistance., I indicates moderate sensitivity, and S indicates sensitivity to antibiotics.

Table S4 The inhibitory effect of CFS on E. coli under different treatment methods

| Processing method | Diameter of inhibition zone/mm |
| --- | --- |
| pH 7.0 | 13.32±0. 10c  20.32±0.26a  17.26±0.53b  20.72±0.39a |
| Catalase |  |
| Protease K |  |
| Control group |  |
